# Exosomes Secreted by Nucleus Pulposus Stem Cells Derived From Degenerative Intervertebral Disc Exacerbate Annulus Fibrosus Cell Degradation *via* Let-7b-5p

**DOI:** 10.3389/fmolb.2021.766115

**Published:** 2022-01-17

**Authors:** Yin Zhuang, Sheng Song, Dan Xiao, Xueguang Liu, Xiaofei Han, Shihao Du, Yuan Li, Yanming He, Shujun Zhang

**Affiliations:** ^1^ Department of Spine Surgery, Wuxi 9th Affiliated Hospital of Soochow University, Wuxi, China; ^2^ Department of Spine Surgery, Orthopedics Center, Guangdong Provincial People’s Hospital, Guangdong Academy of Medical Sciences, Guangzhou, China

**Keywords:** intervertebral disc degeneration, nucleus pulposus stem cells, annulus fibrosus cell, proliferation, apoptosis, miRNA

## Abstract

The pathogenesis of intervertebral disc degeneration (IDD) is complex and remains unclear. Nucleus pulposus stem cells (NPSCs) and annulus fibrosus cells (AFCs) play a critical role in the maintenance of intervertebral disc structure and function. Exosome-mediated miRNAs regulate cell proliferation, differentiation, apoptosis, and degradation. However, it is not clear whether the degenerative intervertebral disc-derived nucleus pulposus stem cells (D-NPSCs) can regulate the function of AFCs by delivering exosomes. Here, we show that exosomes secreted by nucleus pulposus stem cells derived from degenerative intervertebral disc (D-DPSC-exo) can exacerbate AFC degeneration via inhibiting cell proliferation, migration, matrix synthesis, and promoting apoptosis. Specifically, let-7b-5p was highly expressed in D-DPSC-exo. Transfection of let-7b-5p mimic was found to promote apoptosis and inhibit proliferation migration and matrix synthesis of AFCs. In addition, transfection with let-7b-5p inhibitor caused the effect of D-DPSC-exo on AFCs to be reversed. Furthermore, we found that D-DPSC-exo and let-7b-5p inhibited IGF1R expression and blocked the activation of the PI3K–Akt pathway. Results suggested that NPSC-exo exacerbated cell degeneration of AFCs via let-7b-5p, accompanied by inhibition of IGF1R expression, and PI3K–Akt pathway activation. Therefore, insights from this work may provide a clue for targeted molecular therapy of intervertebral disc degeneration.

## Introduction

Intervertebral disc degeneration (IDD) is one of the main causes of lower back pain, but its specific pathogenesis remains unclear (Nakashima et al., 2020). Surgical treatment can relieve symptoms and alleviate pain, but it has no effect on the underlying disease itself (Dowdell et al., 2017). The intervertebral disc is mainly composed of nucleus pulposus cells and annulus fibrosus cells (AFCs), and the senescence and degeneration of these cells are the basis of IDD (Zhang et al., 2019b). Recent advances in strategies to engage stem cell transplantation for IDD therapy have established as a new avenue for the treatment of IDD and achieved many encouraging results (Urits et al., 2019; Vadala et al., 2019), However, there are still many challenges and limitations that need to be further improved, such as short survival time, poor cell activity, and difficulty in differentiation, and the most important issue is that exogenous stem cells cannot tolerate the adverse microenvironment of local hypoxia, hyperosmolarity, low pH, and nutrient deficiency that develops after intervertebral disc degeneration (Wang et al., 2015; Hang et al., 2017; Ma et al., 2019). It may be possible to render the use of stem cells more practical using endogenous nucleus pulposus stem cells (NPSCs) to repair and reconstruct intervertebral disc function, taking advantage of the fact that most adult tissues have their own stem cell niche (Vickers et al., 2019).

NPSCs are mesenchymal stem cells (MSCs) derived from the endogenous nucleus pulposus tissues (Ying et al., 2019). They have the characteristics of self-renewal and differentiation into intervertebral disc cells (Lazzarini et al., 2019). NPSCs can better tolerate the local acidic microenvironment of degenerated disc than other tissue-derived mesenchymal stem cells, and they play an important role in the biological treatment of IDD (Choi et al., 2015). Research has shown that the characteristics of nucleus pulposus stem cells derived from degenerative intervertebral disc (D-DPSCs) and derived from normal intervertebral disc (N-DPSCs) are not exactly the same (Liu et al., 2019). With patient aging and aggravation of intervertebral disc degeneration, the proliferation and stemness of NPSCs decreased, which was not conducive to self-repair of the intervertebral disc (Sakai et al., 2012; Wu et al., 2018). Rather, it was more likely to aggravate the degeneration of the intervertebral disc tissue (Vergroesen et al., 2015).

In addition to direct differentiation into nucleus pulposus cells, the regulation of NPSCs on damaged tissues can also be realized through the paracrine pathway of their secretions (such as exosomes and cytokines) (Schneider and Simons, 2013). Exosomes are lipid bilayer membrane vesicles with diameters of approximately 30–150 nm, which can be secreted by most cell types. Exosomes have the ability to carry proteins, lipids, RNA, and a variety of other biological macromolecules that play an important role in cell-to-cell transfer of materials and information (Du et al., 2017; Liu et al., 2017). Moreover, some studies indicated that MSC-derived exosomes can promote angiogenesis (Aziz et al., 2020) and wound healing (An et al., 2021), as well as regulate immunity (Whiteside, 2018), while aging MSC-derived exosomes do not have this ability. Other studies have shown that aging bone-derived extracellular vesicles inhibited the proliferation of bone marrow stem cells and induced stem cell senescence (Davis et al., 2017).

The effect of NPSCs-derived exosomes on the surrounding tissue cells in the microenvironment of intervertebral disc degeneration is still unknown**.** In this study, we explored the differential effect of exosomes secreted by nucleus pulposus stem cells derived from degenerative intervertebral disc (D-DPSC-exo) and exosomes secreted by nucleus pulposus stem cells derived from normal intervertebral disc (N-DPSC-exo) on annulus fibrosus cells. Results showed that D-DPSC-exo exacerbated AFCs degradation through let-7b-5p by inhibiting the PI3K/AKT signaling pathway.

## Materials and methods

### Cell isolation and culture

All the experimental protocols were approved by the Ethics Committee of Wuxi 9th Affiliated Hospital of Soochow University and were obtained with the informed consent of the patients. Normal nucleus pulposus tissues were obtained from five patients (2 females and 3 males, aged 18–49 years) who underwent spine surgery of burst thoracolumbar fracture and degenerative nucleus pulposus, and the annular fibers tissues were gently obtained from 11 patients (4 females and 7 males, aged 37–61 years) who underwent surgery of disc excision for lumbar degenerative disease. Nucleus pulposus tissues were washed three times with PBS, then cut into pieces, and digested with 1 mg/ml of type II collagenase (Solarbio, China) for 4 h at 37°C. After being filtered through a 70-μm filter, the suspension was centrifuged at 300 × *g* for 5 min, and the isolated cells were cultured in Complete Culture Medium of Mesenchymal Stem Cell (Cyagen, China) containing 88% basal medium, 10% MSC cell-qualified fetal bovine, 1% penicillin-streptomycin, and 1% glutamine. Annular fibers tissues were washed three times with PBS, then cut into pieces, and digested with 1 mg/ml of type I collagenase (Solarbio, China) for 2 h at 37°C. After being filtered through a 70-μm filter, the suspension was centrifuged at 300 × *g* for 5 min, and the isolated cells were cultured in Dulbecco’s Modified Eagle Medium/Nutrient Mixture F-12 (DMEM/F12) (Gibco, United States) containing 10% fetal bovine serum (FBS) (Gemini, China) and 1% penicillin–streptomycin (Gibco, USA). Finally, all the isolated NPSCs and AFCs were incubated at 37°C in a humidified atmosphere of 5% CO_2_ with the culture medium replaced every 3 days.

### Cell identification

The NPSCs were identified by flow cytometry analysis and multipotential differentiation. The multipotential differentiation of NPSCs was determined using an MSCs Adipogenic Differentiation Kit (Cyagen, China) and an MSCs Osteogenic Differentiation Kit (Cyagen, China), respectively. Oil red O staining and Alizarin red staining were used to assess adipogenic and osteogenic differentiation. For the detection of surface markers by flow cytometry, NPSCs were stained for 30 min with fluorescein isothiocyanate (FITC)-conjugated or phycoerythrin (PE)-conjugated anti-human CD29, CD34, CD44, CD45, CD73, CD90, CD105, and HLA-DR monoclonal antibodies, and characterized using flow cytometry (BD Biosciences, USA). The monoclonal antibodies were all purchased from BioLegend, Inc.

The AFCs were identified by immunofluorescence analysis and toluidine blue staining. For the detection of type II collagen immunohistochemical staining, AFCs were incubated with primary antibody type II collagen (1:200, Abcam, USA) at 4°C overnight. After PBS washing three times, cells were incubated with FITC-conjugated goat-anti-rabbit secondary antibody (1:1,000, Beyotime, China) at room temperature for 1 h before counterstaining with DAPI (Solarbio, China). Images were obtained using a fluorescence microscopy (Olympus, Japan). For toluidine blue staining, AFCs stained with 1% toluidine blue for 30 min at room temperature before fixing in 4% paraformaldehyde for 30 min, then briefly rinsed in ethanol and observed using an inverted biological microscope (Olympus, Japan).

### Exosome isolation, purification, and identification

N-NPSCs and D-NPSCs were cultured in exosome-free medium for 2 days. NPSC-exo was isolated from supernatant by ultracentrifugation. The culture medium was centrifuged at 1,000 × *g* for 10 min at 4°C to remove dead cells and centrifuged at 10,000 × *g* for 30 min at 4°C to remove cell debris. Next, exosomes were centrifuged by ultracentrifugation at 100,000 × *g* for 70 min after filtering through 0.22-μm membrane filters. Moreover, exosomes were purified by a commercial kit using ExoJuice (WeinaBio, China) according to the instruction of the manufacturer. Briefly, exosome samples were transferred to 5-ml ultracentrifuge tubes and 500 µl of Exojuice was added to the bottom. The tube was then centrifuged at 100,000 × *g* for 70 min at 4°C, carefully recovered, and fractionated from the bottom. The first 200 µl of the liquid from the bottom of the tube was discarded, then the next 200 µl fraction of solution from the bottom was collected, which contained the purified exosomes.

After purification, morphology was observed by transmission electron microscopy, particle diameter and concentration were analyzed by flow nano analysis, and the exosomal markers, such as CD9, CD81, and TSG101 (Affinity, USA) were detected by Western blotting assay.

### Cell counting kit-8 assay

Cell counting kit-8 (CCK-8) assay was used to value the proliferation capacity of AFCs. Briefly, AFCs were plated in 96-well plates at a density of 5 × 10^3^ cells per well with 100 μl of complete culture medium. After 24 h, cells were treated with exosomes (or miRNA mimic/inhibitor) and incubated for 48 h, respectively. Finally, 10 μl of CCK-8 reagents (APExBIO, USA) was added to each well and then incubated for another 2 h at 37°C. The absorbance value at 450 nm was detected by a microplate reader (Biorad imark, USA).

### Transwell assay

Transwell assay was used to value the migration capacity of AFCs. Briefly, AFCs (5 × 10^4^ cells per well) in 100 μl of serum-free medium was transferred to the upper chambers of the Transwell, then 600 μl of medium treated with exosomes (or miRNA mimic/inhibitor) was added to the bottom chambers, as previously described, respectively. After 24 h, cells were fixed in 4% paraformaldehyde and stained with 0.1% crystal violet. Then the cells were observed and the stained migrated cells were counted by using a microscope.

### Western blotting

Exosomes or cells were lysed with RIPA buffer, then the protein concentration was measured using the BCA protein detection kit (Beyotime, China). Each sample was mixed with protein loading buffer (5×) and heated at 95°C for 10 min to denature. Then protein samples were separated by 10% SDS–polyacrylamide gel electrophoresis and transferred to polyvinylidene fluoride membranes (PVDF) (Millipore, Germany). PVDF membranes were blocked with 5% skim milk for 1 h, and primary antibody (MMP-13, collagenase II, IGFR1, p-PI3K, *p*-AKT, GAPDH, and β-ACTIN) (Bioss, China) was added in for incubating overnight at 4°C, then the secondary antibody conjugated with horseradish peroxidase was incubated for 2 h at room temperature. Fluorescence was analyzed using the enhanced chemiluminescence kit (Tanon, China) and imaged by the luminescent image analyzer (Tanon, China), and the absorbance value of each band was calculated using the ImageJ software.

### Flow cytometry analysis

Apoptosis rates were evaluated using an Annexin V/PI apoptosis detection kit (BD Biosciences, USA) according to the instruction of the manufacturer. Annexin V-FITC (5 μl) and PI (5 μl) solutions were added into 100 μl of cell suspension, respectively. The cells were incubated at room temperature for 15 min in the dark, cells were detected, and analyzed by flow cytometry (Beckman, USA).

### miRNA sequencing

Exosomal miRNAs were sequenced in N-NPSC-exo and D-NPSC-exo. The total RNA was isolated from the exosomes using TRIzol reagent (Life Technologies, United States) according to the instructions of the manufacturer. The extracted RNA was then quantified using the OneDrop (WuYi, China). The sample quality control, library preparation, and sequencing were performed by HuaYing, China.

### Analysis of the effects of miRNA mimics and inhibitors

According to the results of the miRNA sequencing of exosomes, the mimics and inhibitors of let-7b-5p and the control were synthesized by WeinaBio (Foshan, China).

AFCs were transfected with miRNA mimics or control at 100 nM with Lipofectamine 3,000 reagent (Invitrogen, USA) and then cultured for 48 h. To further confirm the effects of miRNAs in exosomes, AFCs were cultured in medium with D-NPSC-exo (50 μg/ml) and then were transfected with miRNA inhibitors (100 nM) or control for 48 h. Cell proliferation, migration, and apoptosis was detected by CCK-8 assay, Transwell assay, and flow cytometry analysis, and the relative proteins were detected by Western blotting.

### qRT-PCR assay

The total RNA was isolated from the exosomes and cells by using TRIzol reagent (Life Technologies, USA). For miRNA, the first-strand cDNA was synthesized by using miRNA 1st Strand cDNA Synthesis Kit (Vazyme, China), then real-time PCR was performed by miRNA Universal SYBR qPCR Master Mix (Vazyme, China) under LightCycler480 Real-Time PCR Detection System (Roche, Switzerland). For mRNA, the first-strand cDNA was synthesized by using HiScript III 1st Strand cDNA Synthesis Kit (Vazyme, China), then real-time PCR was performed by Universal SYBR qPCR Master Mix (Vazyme, China). U6 was used as an internal reference for miRNAs, and beta-actin was for mRNAs. Comparative quantification was determined using the 2^−ΔΔCt^ method. The primer sequences used are summarized in Supplementary Table S1.

### Statistical analysis

GraphPad Prism 8 software was used for statistical analysis. Data are expressed as mean ± standard deviation. The differences between the two groups were analyzed with the unpaired *t*-test. A *p-*value < 0.05 was considered statistically significant.

## Results

### Cell identification

**FIGURE 1:**
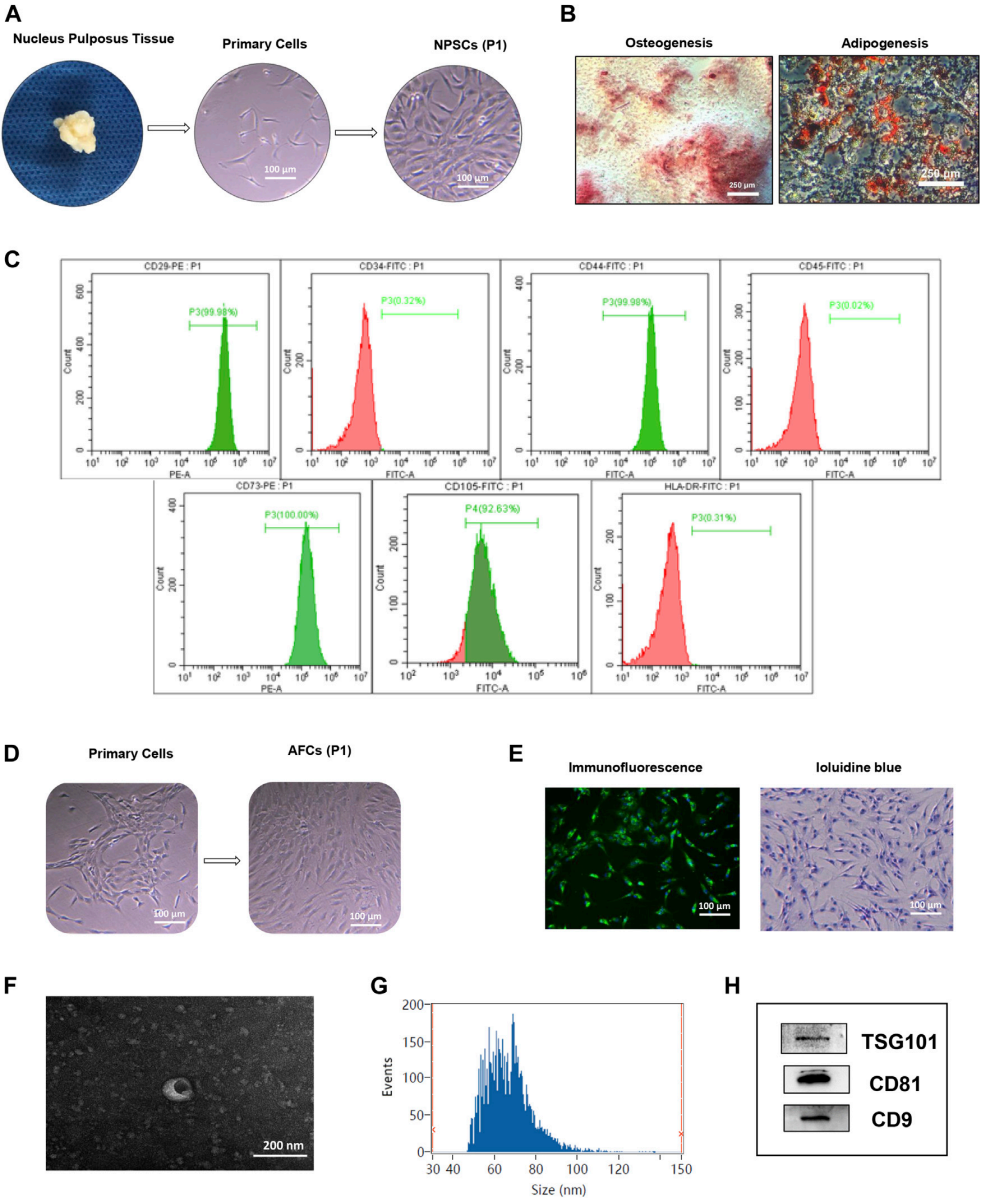
Identification of nucleus pulposus stem cells (NPSCs), annulus fibrosus cells (AFCs), and NPSC-exo. **(A)** Process of isolation and culture of NPSCs from nucleus pulposus tissue. Cultured NPSCs at passage 1 (P1) were spindle shaped. **(B)** Differentiation potential of osteogenesis and adipogenesis of NPSCs was confirmed by Alizarin red staining and Oil red O staining after 2 weeks after *in vitro* induction, respectively. **(C)** Identification of surface makers of NPSCs indicated that the harvested NPSCs were positive expressions for CD29, CD44, CD73, and CD105 makers, and were negative expressions for CD34, CD45, and HLA-DR makers. **(D)** The cell morphology of cultured AFCs at passage 1 (P1) displayed long fusiform or polygonal shaped. **(E)** Immunofluorescence staining indicated that AFCs expressed type II collagen and toluidine blue staining showed AFCs were purple and exhibited metachromatic granules. **(F)** The typical saucer-like morphology of NPSC-exo was captured by transmission electron microscopy. **(G)** Particle size of NPSC-exo was characterized by flow nano analysis. **(H)** NPSC-exo were positive expressions for exosomal surface markers TSG101, CD81, and CD9 by Western blotting.

Isolated NPSCs displayed fibroblast-like morphology and vortex-styled adherent growth in culture (Figure 1A). The results of induced differentiation *in vitro* showed visible calcium deposits formed and stained bright red by Alizarin red staining after osteogenic differentiation, and lipid droplets appeared and stained red by Oil red O staining after adipogenic differentiation (Figure 1B). Based on the flow cytometry analysis, NPSCs were positive for surface markers CD29, CD44, CD73, and CD105 (>90%), and negative for surface markers CD34, CD45, and HLA-DR (<2%) (Figure 1C). AFCs displayed a long spindle or polygon shape (Figure 1D), and expressed type II collagen by immunofluorescence staining (Figure 1E left panel). The toluidine blue staining showed AFCs were purple and exhibited metachromatic granules (Figure 1E right panel).

### Exosome identification

Morphology, diameter, and surface markers of NPSC-exo were characterized by transmission electron microscopy, flow nano analysis, and Western blotting, respectively. NPSC-exo was displayed as cup-shaped vesicles using transmission electron microscopy (Figure 1F). The mean diameter of the exosomes was 66.64 nm, and the concentration of exosomes was 8.8 × 10^9^ particles/ml using flow nano analysis (Figure 1G). Western blotting results showed that NPSC-exo were positive for exosomal surface markers TSG101, CD81, and CD9 (Figure 1H).

### Effect of N-NPSC-Exo and D-NPSC-Exo on cell proliferation, migration, apoptosis, and extracellular matrix metabolism

**FIGURE 2:**
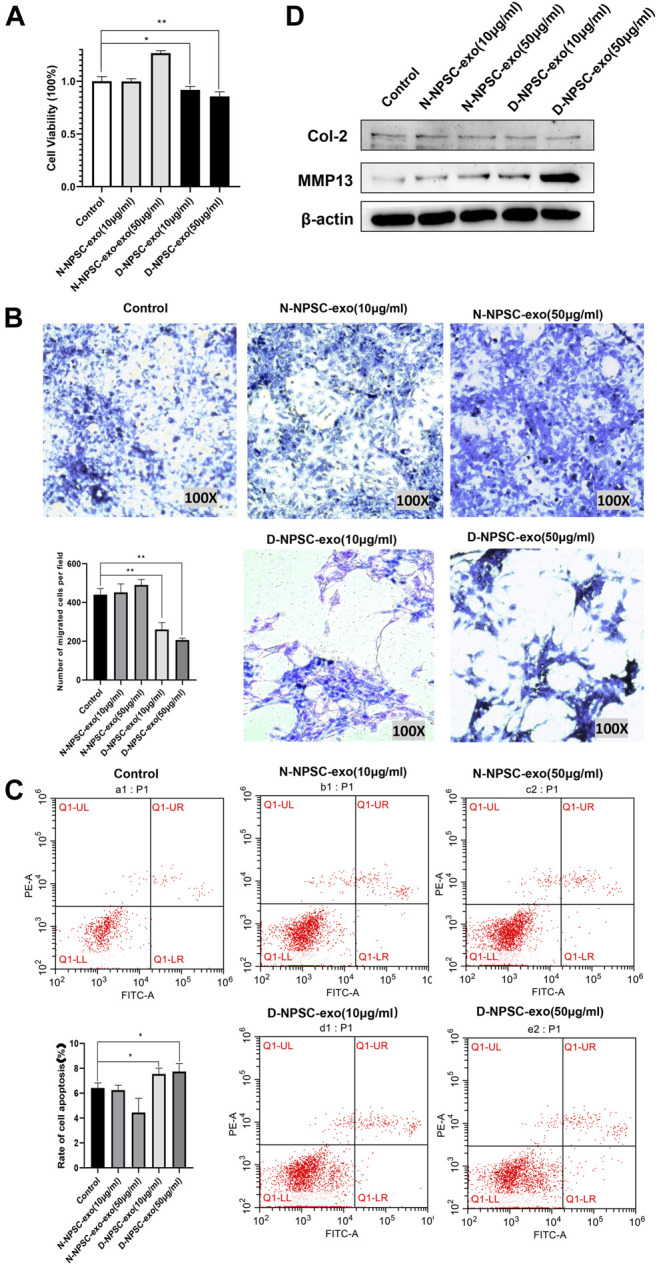
Degenerative intervertebral disc-derived nucleus pulposus stem cells secreted exosomes (D-NPSC-exo) inhibited proliferation, migration, and extracellular matrix synthesis, and promoted apoptosis of AFCs. **(A)** Proliferation of AFCs treated with D-NPSC-exo, normal intervertebral disc-derived nucleus pulposus stem cells secreted exosomes (N-NPSC-exo), or PBS (control group) was evaluated by cell counting kit 8 (CCK-8) assay. D-NPSC-exo in both low (10 μg/ml) and high (50 μg/ml) concentrations significantly inhibited cell proliferation, and N-NPSC-exo in high (50 μg/ml) concentrations promoted cell proliferation, oppositely. **(B)** Migration of AFCs was evaluated by Transwell assay. D-NPSC-exo in both low (10 μg/ml) and high (50 μg/ml) concentrations significantly inhibited cell migration, and N-NPSC-exo promoted cell migration, oppositely. **(C)** Apoptosis of AFCs was evaluated by Annexin V/PI apoptosis detection kit. D-NPSC-exo in both low (10 μg/ml) and high (50 μg/ml) concentrations significantly promoted cell apoptosis. However, N-NPSC-exo in high (50 μg/ml) concentrations reduced the apoptotic rate of AFCs. **(D)** Protein expression levels of Col II, and MMP13 were detected by Western blotting. D-NPSC-exo in high (50 μg/ml) concentrations significantly promoted the expression of MMP13 and inhibits the expression of Col II. *p < 0.05; **p < 0.01.

To explore the effect of the NPSC-exo in AFCs, N-NPSC-exo and D-NPSC-exo in concentrations of 10 and 50 μg/ml were incubated with AFCs, respectively. Then cell proliferation, migration, apoptosis, and extracellular matrix metabolism were analyzed using CCK-8 assay, Transwell migration assay, flow cytometry, and Western blotting, respectively. Our results showed that D-NPSC-exo in low and high concentrations also significantly inhibited cell proliferation (Figure 2A), suppressed cell migration (Figure 2B), and promoted the apoptosis of AFCs (Figure 2C). On the contrary, N-NPSC-exo on high concentration tended to promote cell proliferation and migration, and inhibit cell apoptosis. In order to detect the effect of NPSC-exo on extracellular matrix metabolism in AFCs, protein expression levels of Col II, and MMP13 were detected by Western blotting. D-NPSC-exo in high (50 μg/ml) concentrations significantly promoted the expression of MMP13 and inhibits the expression of Col II. However, the N-NPSC-exo had no effect on extracellular matrix secretion in AFCs (Figure 2D).

### Differentially expressed miRNAs in N-NPSC-Exo and D-NPSC-Exo

**FIGURE 3:**
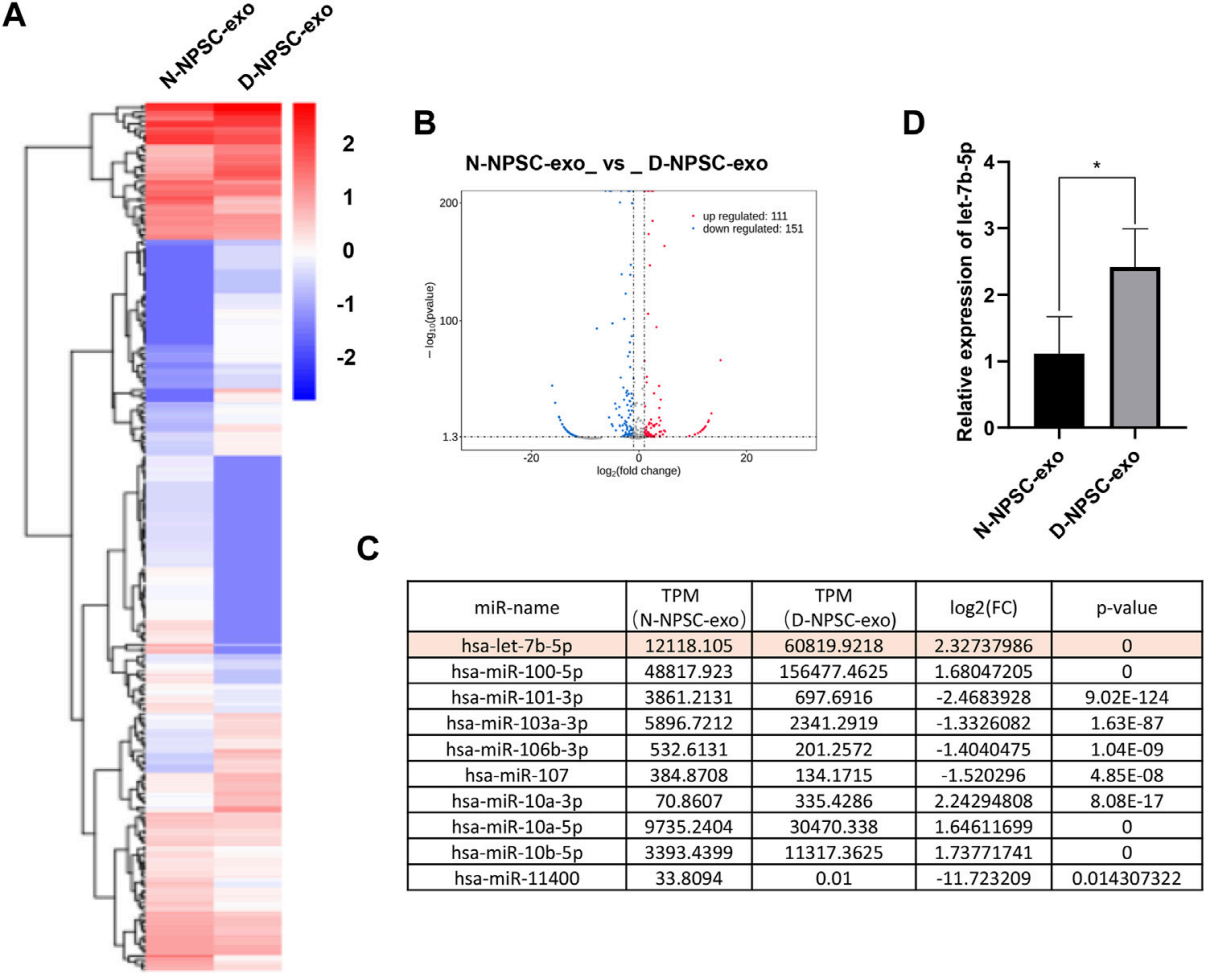
Profiling of the expression of miRNA in N-NPSC-exo and D-NPSC-exo. **(A)** The miRNA profiles of N-NPSC-exo and D-NPSC-exo are represented in the heat map and hierarchical clustering-based dendrograms. **(B)** Volcano plot showing up- and downregulated miRNAs in N-NPSC-exo compared with D-NPSC-exo. **(C)** Fold changes observed in miRNA expression in N-NPSC-exo compared with D-NPSC-exo on microarray data. **(D)** Real-time PCR of hsa-let-7b-5p in N-NPSC-exo compared with D-NPSC-exo. *p < 0.05; **p < 0.01.

To detect the different miRNA constituents of N-NPSC-exo and D-NPSC-exo, miRNA sequencing was conducted. A clustered heat map (Figure 3A) shows the upregulated and downregulated miRNA expressions, and a volcano plot (Figure 3B) shows log 2 (fold change) (N-NPSC-exo vs. D-NPSC-exo) on the *x*-axis and −log 10 (*p-*value) on the *y*-axis. Ten significantly up-/downregulated miRNAs in N-NPSC-exo compared with D-NPSC-exo were listed (Figure 3C), and let-7b-5p was upregulated in D-NPSC-exo on the microarray data. Consistent with the sequencing results, the expression of let-7b-5p was upregulated in D-NPSC-exo compared with N-NPSC-exo by real-time PCR analysis (Figure 3D).

### Overexpression of let-7b-5p inhibited proliferation, migration, and extracellular matrix synthesis, and promoted apoptosis of annulus fibrosus cells

**FIGURE 4:**
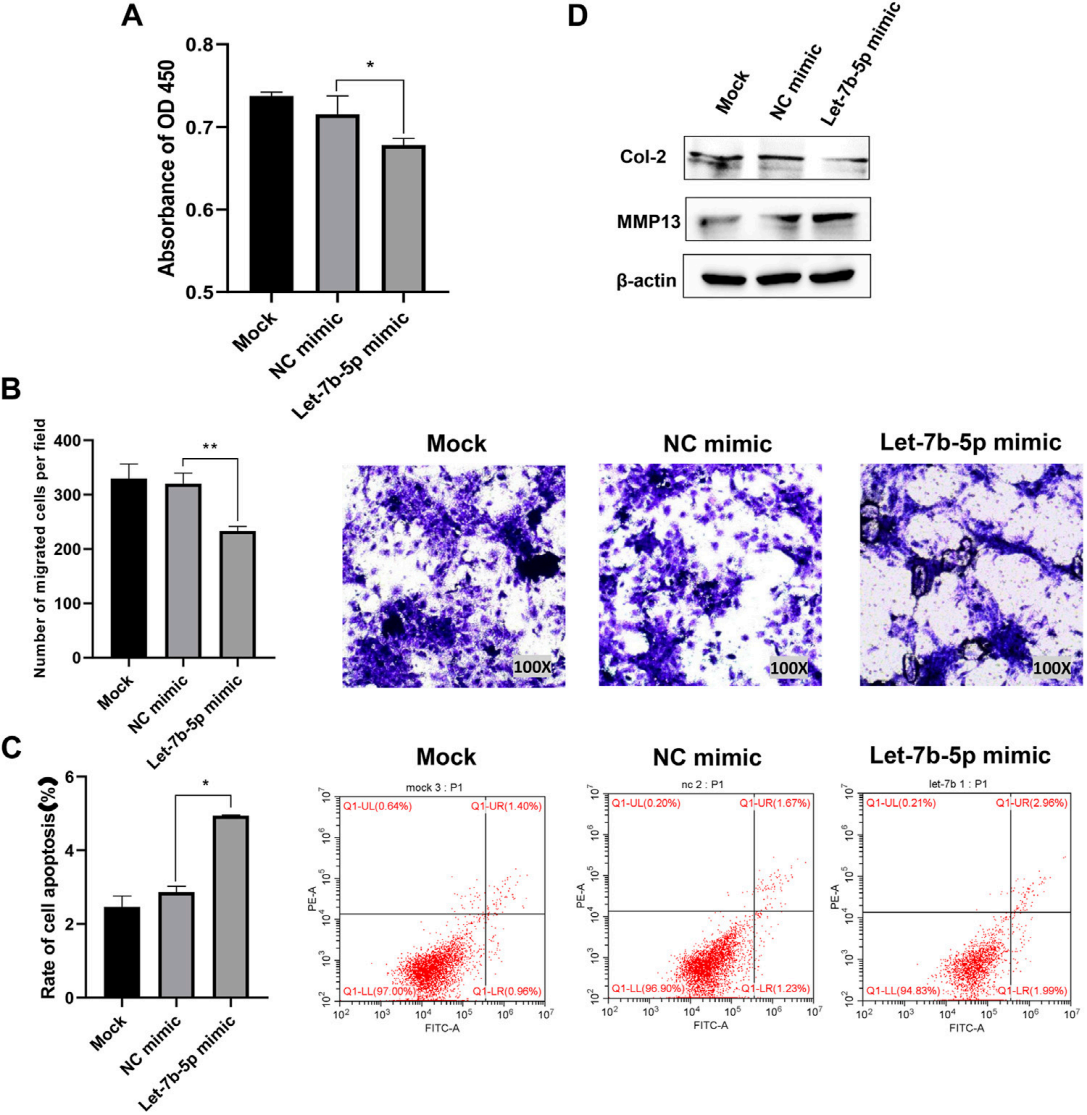
Overexpression of let-7b-5p inhibited proliferation, migration, and extracellular matrix synthesis, and promoted apoptosis of AFCs. AFCs were transfected with let-7b-5p mimic, NC mimic, or PBS (mock group). **(A)** Let-7b-5p mimic inhibited cell proliferation of AFCs by CCK-8 assay. **(B)** Let-7b-5p mimic inhibited cell migration of AFCs by Transwell assay. **(C)** Let-7b-5p mimic promoted cell apoptosis of AFCs by Annexin V/PI apoptosis detection kit. **(D)** Let-7b-5p mimic promoted the expression of MMP13 and inhibits the expression of Col II by Western blotting. *p < 0.05; **p < 0.01.

The results of CCK-8 assay and transwell assay showed that overexpression of let-7b-5p by transfecting with let-7b-5p mimic significantly inhibited proliferation (Figure 4A) and migration (Figure 4B) of AFCs. The transfection of let-7b-5p mimic significantly promoted the apoptotic ratio compared with the NC mimic by Annexin V/PI apoptosis detection kit (Figure 4C). Moreover, the decreased protein level of Col II and elevated MMP13 expression demonstrated that overexpression of let-7b-5p mimic affected the synthesis of the extracellular matrix (Figure 4D).

### Inhibition of let-7b-5p in D-NPSC-Exo alleviated cell degeneration of annulus fibrosus cells

**FIGURE 5:**
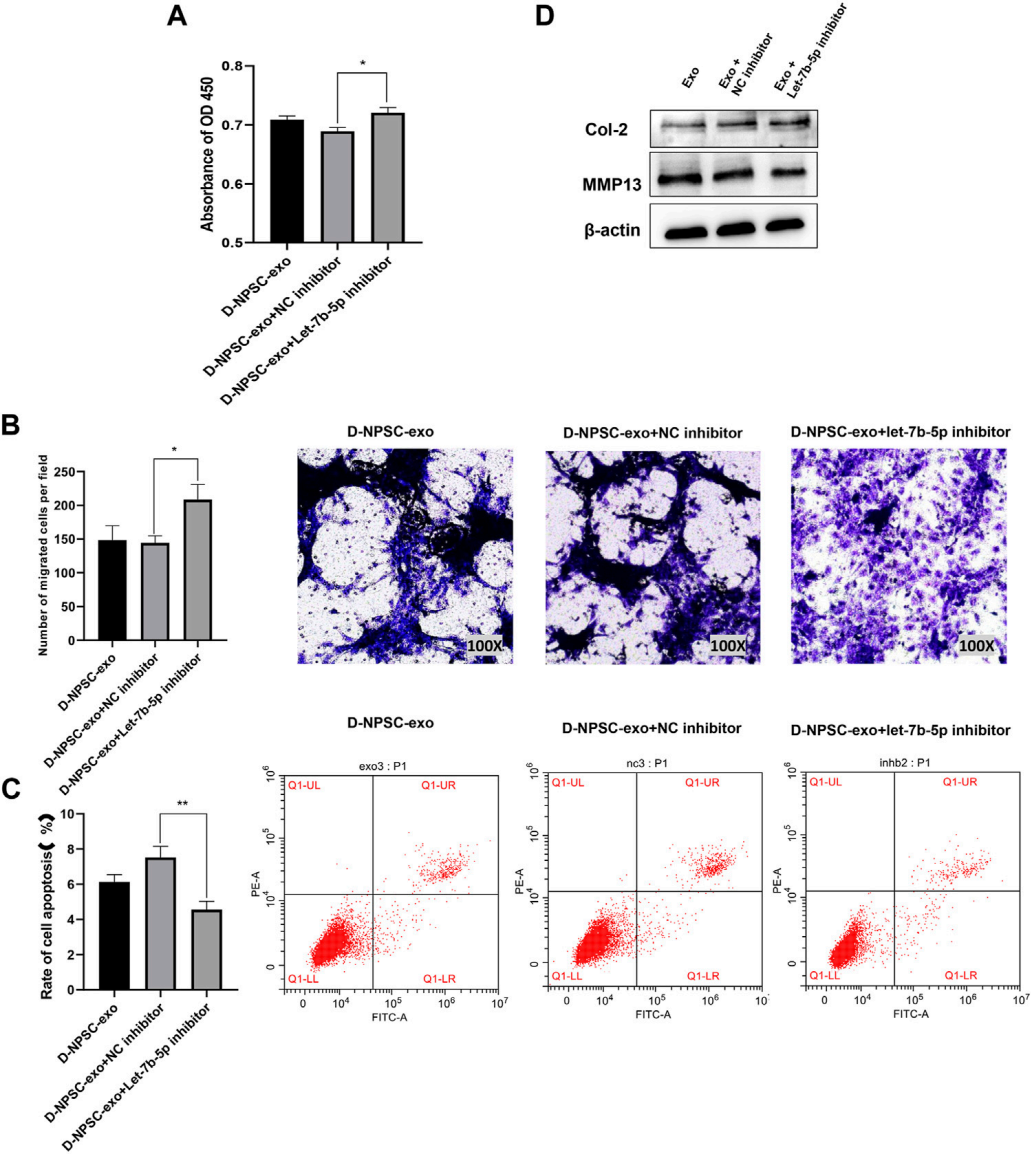
Inhibition of let-7b-5p in D-NPSC-exo alleviated cell degeneration of AFCs. AFCs were treated with D-NPSC-exo, then transfected with let-7b-5p inhibitor or NC inhibitor. **(A)** Inhibition of exosomal let-7b-5p promoted cell proliferation of AFCs by CCK-8 assay. **(B)** Inhibition of exosomal let-7b-5p promoted cell migration of AFCs by Transwell assay. **(C)** Inhibition of exosomal let-7b-5p inhibited cell apoptosis of AFCs by Annexin V/PI apoptosis detection kit. **(D)** Inhibition of exosomal let-7b-5p inhibits the expression of MMP13 and promoted the expression of Col II by Western blotting. *p < 0.05; **p < 0.01.

To further confirm that D-NPSC-exo inhibited proliferation, migration, and extracellular matrix synthesis, and promoted apoptosis of AFCs by delivering let-7b-5p, the let-7b-5p inhibitor was applied to transfect with D-NPSC-exo-treated AFCs. The overall effects of D-NPSC-exo on AFCs were abolished by the let-7b-5p inhibitor. Compared with the NC inhibitor groups, the cell proliferation (Figure 5A) and migration (Figure 5B) of AFCs were promoted, and cell apoptosis (Figure 5C) of AFCs was reduced. Meanwhile, the protein expression of MMP13 was reduced, and the protein expression of Col II was promoted (Figure 5D). Collectively, exosomal let-7b-5p plays important roles in cell degeneration of AFCs.

### Exosomal let-7b-5p leads to targeted inhibition of IGF1R followed by inhibiting the activation of the PI3K/Akt pathway in annulus fibrosus cells

**FIGURE 6:**
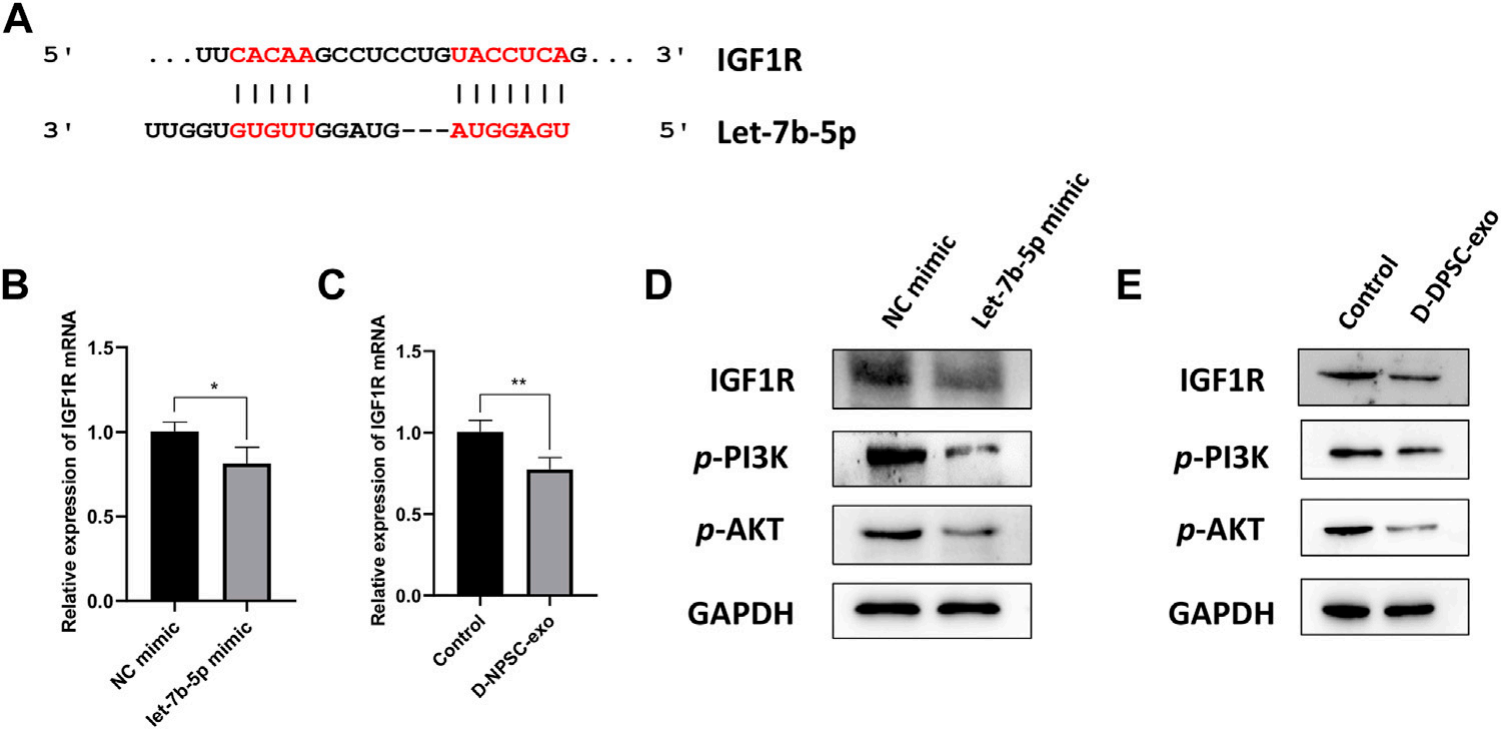
D-NPSC-exo aggravated cell degeneration of AFCs by inhibiting the activation of the IGF1R/PI3K/Akt pathway. **(A)** Shown are let-7b-5p binding sites in the IGF1R 3′ UTR as predicted in TargetScan. **(B)** mRNA expression level of IGF1R in AFCs transfected with let-7b-5p mimic was detected by real-time PCR. **(C)** mRNA expression level of IGF1R in AFCs treated with D-NPSC-exo was detected by real-time PCR. **(D)** Protein expression levels of IGF1R, *p*-PI3K, and *p*-AKT in AFCs transfected with let-7b-5p mimic were detected by Western blotting. **(E)** Protein expression levels of IGF1R, *p*-PI3K, and *p*-AKT in AFCs treated with D-NPSC-exo were detected by Western blotting. *p < 0.05; **p < 0.01.

Finally, we explored the mechanism by which exosomal let-7b-5p aggravated cell degeneration of AFCs. A binding site at 3′-UTR of IGF1R was predicted on let-7b-5p by TargetScan (Figure 6A). The result of real-time PCR confirmed that the mRNA expression of IGF1R was significantly decreased in AFCs by transfecting with let-7b-5p (Figure 6B). Similarly, this decrease in the mRNA expression of IGF1R appeared in D-NPSC-exo-treated AFCs (Figure 6C). To test whether exosomal let-7b-5p modulates IGF1R and the PI3K/Akt signal pathway, protein expression levels of IGF1R, *p*-PI3K, and *p*-AKT in AFCs transfected with let-7b-5p mimic were detected by Western blotting. The result of Western blotting confirmed that the protein expressions of IGF1R, *p*-PI3K, and *p*-AKT were decreased in AFCs by transfecting with let-7b-5p (Figure 6D). Similarly, this decrease in the protein expressions of IGF1R, p-PI3K, and *p*-AKT appeared in D-NPSC-exo-treated AFCs (Figure 6E). Collectively, exosomal let-7b-5p leads to targeted inhibition of IGF1R followed by inhibiting the activation of the PI3K/Akt pathway in AFCs.

## Discussion

In recent years, stem cell transplantation has become a research hotspot for biological treatment of IDD (Xia et al., 2019). Some studies found that transplantation of autologous NPSCs into degenerative intervertebral disc tissues can significantly delay the development of IDD (Chen et al., 2016). NPSCs are progenitors of nucleus pulposus cells, and they have considerable potential for proliferation and differentiation, and some results have been observed in both normal and degenerative intervertebral disc tissues. Endogenous NPSCs can better tolerate the local acidic and hypertonic microenvironment and play a key role in stem cell biological treatment of IDD (Tao et al., 2013; Han et al., 2014). However, Sakai found that the number of NPSCs in the nucleus pulposus gradually decreased with aging and the degree of intervertebral disc degeneration in rats and humans. Sakai also pointed out that the intervertebral disc degeneration may be caused by the depletion of NPSC apoptosis (Sakai et al., 2012). A study by Liu showed that the biological characteristics of NPSCs derived from normal and degenerative intervertebral discs were different (Liu et al., 2019).

Currently, researchers believe that secreting exosomes is one of the important ways in which stem cells perform biological functions (Phinney and Pittenger, 2017; Mendt et al., 2019). To determine whether there are functional differences between N-NPSC-exo and D-NPSC-exo, we studied the effects of N-NPSC-exo and D-NPSC-exo on the proliferation, migration, apoptosis, and extracellular matrix synthesis of degenerated AFCs. The results showed that N-NPSC-exo was beneficial to AFCs. However, D-NPSC-exo inhibited AFC proliferation, migration, and matrix synthesis, and promoted apoptosis.

miRNAs, important intermediaries of the exosome function, play an important role in maintaining normal homeostasis (Shan et al., 2019). Exosomes transport miRNAs to recipient cells and participate in such physiological processes as cell proliferation, differentiation, migration, and apoptosis (Zhang et al., 2020). To further explore how D-NPSC-exo affects AFCs, N-NPSC-exo and D-NPSC-exo were sequenced and found to be discrepant in some miRNAs. This difference may regulate the cellular function of AFCs. We found that let-7b-5p was upregulated in D-NPSC-exo, and was confirmed by real-time PCR, which suggested a possible connection between the increase of miRNA and the AFCs degeneration. The mimic and inhibitor of let-7b-5p were used to establish the effects of miRNA in D-DPSC-exo. After transfection with the let-7b-5p mimic, the proliferation, migration, and extracellular matrix synthesis were reduced, and the rate of apoptosis among AFCs increased significantly. After transfection with the let-7b-5p inhibitor, the effect of D-DPSC-exo on AFCs was reversed. This indicated that D-DPSC-exo may exacerbate AFC degeneration by delivering let-7b-5p.

Let-7b-5p is a miRNA with a wide range of biological functions (Mandolesi et al., 2021). It plays a very important role in cell migration and proliferation and in antitumor processes (Babapoor et al., 2017; Wu et al., 2020). Let-7b-5p has been shown to regulate proliferation and apoptosis in multiple myeloma by targeting IGF1R (Xu et al., 2014). The results of the study of Zhang indicated that let-7b suppressed proliferation and invasion of osteosarcoma cells via targeting IGF1R (Zhang et al., 2019a). In this present work, real-time PCR and Western blotting analysis confirmed that the mRNA and protein expression of IGF1R were significantly decreased in AFCs by transfecting with let-7b-5p.

Many studies confirmed that IGF1R is one of the upstream regulatory molecules of the PI3K-Akt pathway, involved in cell proliferation, apoptosis, and metabolism (Park et al., 2018). Here, we confirmed that the activation of the PI3K/Akt pathway could be blocked by the let-7b-5p mimic targeting IGF1R. Our results also suggested that D-NPSC-exo exacerbated cell degeneration of AFCs *via* let-7b-5p, possibly by blocking the IGF1R/PI3K/Akt pathway.

## Conclusion

In summary, let-7b-5p carried by D-NPSC-exo was found to regulate the function of AFCs by downregulating IGF1R and blocking the PI3K/Akt pathway, thus, ultimately exacerbating cell degeneration of AFCs. These results suggest the clinical application prospect of D-NPSC-exo-derived let-7b-5p, possibly as a molecular target in the treatment of IDD.

## Data Availability

The data and materials used to support the findings of this experiment are available from the corresponding author upon reasonable request. The raw data has been deposited at: https://www.aliyundrive.com/s/nnbQQNG2ZQm.
