# Supplementary material for: Exosomes Secreted by Nucleus Pulposus Stem Cells Derived From Degenerative Intervertebral Disc Exacerbate Annulus Fibrosus Cell Degradation via Let-7b-5p
Source: Front Mol Biosci. 2022 Jan 17;8:766115. doi: 10.3389/fmolb.2021.766115 (PMC8802296; doi:10.3389/fmolb.2021.766115)
Supplement: Supplementary file 1 [file Table1.DOCX]

**Supplementary Table S1. List of specific primers.**

| **Gene** | **Sequence** |
| --- | --- |
| *Let-7b-5p* | RT: 5’-GTCGTATCCAGTGCAGGGTCCGAGGTATTCGCACTGGATACGACACCAC-3’ |
|  | F: 5’-CGCGCGTGAGGTAGTAGGTT-3’ |
|  | R: 5’-AGTGCAGGGTCCGAGGTATT-3’ |
| *U6* | F: 5’-GTGCTCGCTTCGGCAGCAC-3’ |
|  | R: 5’-AAAATATGGAACGCTTCACGAATT-3’ |
| *IFG1R* | F: 5’-TGCTGACCTCTGTTACCTCTCCAC-3’ |
|  | R: 5’-GTCTTCTCACACATCGGCTTCTCC-3’ |
| *β-actin* | F: 5’-GGCATCCACGAAACTACATTCAATTCC-3’ |
|  | R: 5’-GTACCACCAGACAGCACTGTGTTG-3’ |

RT: reverse transcription F: [forward](javascript:;) R:[reverse](javascript:;)
